# Altered RNA export by SF3B1 mutants confers sensitivity to nuclear export inhibition

**DOI:** 10.1038/s41375-024-02328-1

**Published:** 2024-07-13

**Authors:** Sana Chaudhry, Felipe Beckedorff, Shaista Shabbir Jasdanwala, Tulasigeri M. Totiger, Maurizio Affer, Abimbola Eunice Lawal, Skye Montoya, Francesco Tamiro, Olivia Tonini, Alexandra Chirino, Andrew Adams, Anya K. Sondhi, Stephen Noudali, Alyssa Mauri Cornista, Miah Nicholls, Jumana Afaghani, Paola Robayo, Daniel Bilbao, Stephen D. Nimer, Jose Antonio Rodríguez, Shruti Bhatt, Eric Wang, Justin Taylor

**Affiliations:** 1 Sylvester Comprehensive Cancer Center at the University of Miami Miller School of Medicine, Miami, FL USA; 2 Department of Human Genetics, University of Miami Miller School of Medicine, Miami, FL USA; 3 Department of Pharmacy and Pharmaceutical Sciences, National University of Singapore, Singapore, Singapore; 4 The Jackson Laboratory for Genomic Medicine, Farmington, CT USA; 5 Department of Pathology and Laboratory Medicine, University of Miami Miller School of Medicine, Miami, FL USA; 6 Department of Medicine, University of Miami Miller School of Medicine, Miami, FL USA; 7 Department of Genetics, Physical Anthropology and Animal Physiology, University of the Basque Country (UPV/EHU), Leioa, Spain

**Keywords:** Myelodysplastic syndrome, Apoptosis, Targeted therapies

## Abstract

SF3B1 mutations frequently occur in cancer yet lack targeted therapies. Clinical trials of XPO1 inhibitors, selinexor and eltanexor, in high-risk myelodysplastic neoplasms (MDS) revealed responders were enriched with SF3B1 mutations. Given that XPO1 (Exportin-1) is a nuclear exporter responsible for the export of proteins and multiple RNA species, this led to the hypothesis that SF3B1-mutant cells are sensitive to XPO1 inhibition, potentially due to altered splicing. Subsequent RNA sequencing after XPO1 inhibition in SF3B1 wildtype and mutant cells showed increased nuclear retention of RNA transcripts and increased alternative splicing in the SF3B1 mutant cells particularly of genes that impact apoptotic pathways. To identify novel drug combinations that synergize with XPO1 inhibition, a forward genetic screen was performed with eltanexor treatment implicating anti-apoptotic targets BCL2 and BCLXL, which were validated by functional testing in vitro and in vivo. These targets were tested in vivo using *Sf3b1*^K700E^ conditional knock-in mice, which showed that the combination of eltanexor and venetoclax (BCL2 inhibitor) had a preferential sensitivity for SF3B1 mutant cells without excessive toxicity. In this study, we unveil the mechanisms underlying sensitization to XPO1 inhibition in SF3B1-mutant MDS and preclinically rationalize the combination of eltanexor and venetoclax for high-risk MDS.

## Introduction

Myelodysplastic neoplasms (MDS) represent a heterogeneous group of clonal hematologic malignancies characterized by ineffective hematopoiesis, cytopenia, and the risk of transformation to acute myeloid leukemia (AML) [1–3]. Despite significant advances in our understanding of MDS, there is no curative treatment beyond an allogeneic hematopoietic stem cell transplant (HSCT) [4, 5]. Since MDS typically affects individuals in their 7th and 8th decades of life, HSCT is not an option for most patients, and the standard of care for high-risk MDS patients ineligible for HSCT are hypomethylating agents azacitidine or decitabine [6, 7]. There are currently no standard treatment options for patients with high-risk MDS refractory to hypomethylating agents, and the median survival for these patients is less than six months [8–10].

Nucleocytoplasmic transport of molecules is an essential process in maintaining cellular homeostasis and has recently been recognized as a therapeutic vulnerability in cancer [11]. Exportin-1 or XPO1 is involved in the export of over 200 proteins bearing the nuclear export signal (NES) and various RNA species, including messenger RNAs and small nuclear RNAs from the nucleus into the cytoplasm [12–14]. Thus, XPO1 is involved in many cellular processes, including gene expression, apoptosis, and cell cycle regulation [15]. The overexpression of XPO1 has been identified in various cancers, including MDS, and is associated with a poor prognosis [16–20]. Selective inhibitors of XPO1 (XPO1i), such as selinexor and eltanexor, have been developed to target the activity of XPO1 [21]. Selinexor is a reversible XPO1 inhibitor that has been FDA-approved for the treatment of multiple myeloma [22] and diffuse large B-cell lymphoma [23]. Eltanexor is a second-generation XPO1i designed to be more tolerable in patients and was recently granted orphan drug designation for MDS; however, the development and understanding of the optimal use of XPO1i in MDS is incomplete [24]. Identifying potential subgroups of MDS that are more sensitive to XPO1i is crucial to optimizing the safety and efficacy of XPO1 inhibition in MDS patients.

Mutations in the splicing factor 3B subunit 1 (*SF3B1*) gene are the most commonly mutated spliceosome gene, occurring in 25–30% of patients with MDS [25] and 2–5% of patients with acute myeloid leukemia (AML) [26]. SF3B1 plays a critical role in the assembly of the spliceosome, the machinery responsible for RNA splicing [27–29]. SF3B1 mutations lead to the development of MDS; however, the functional consequences of SF3B1 mutations and how their deregulation of splicing leads to oncogenesis is not completely understood [30, 31]. We recently completed a phase 2 clinical trial of selinexor in patients with MDS relapsed or refractory to hypomethylating agents and found that the presence of a hotspot mutation in *SF3B1* was associated with response to selinexor [32]. Given that XPO1 is required for the nuclear export of RNA components of the spliceosome and that *SF3B1* mutations increased sensitivity to XPO1i, here we determined the mechanistic link between *SF3B1* mutations and XPO1i sensitivity in MDS. Furthermore, we used forward genetic screens to identify combinations of anti-apoptotic drugs that synergize with XPO1i in vitro and in vivo to identify therapeutic combinations with greater efficacy in MDS patients.

## Methods

### Cell lines

K562 (RRID: CVCL_0004) cells were maintained in IMDM media with 10% fetal bovine serum (FBS) and 1% penicillin-streptomycin (P/S). The leukemia cell lines NALM6 (RRID: CVCL_0092), MOLM13 (RRID: CVCL_2119), and U937 (RRID: CVCL_0007) were maintained in RPMI-1640 media supplemented with 10% FBS and 1% P/S. Cell lines were tested for Mycoplasma using the MycoAlert Mycoplasma Detection Kit (Lonza). Validation of the *SF3B1* mutation was routinely performed by PCR and Sanger sequencing. Venetoclax (HY-15531), navitoclax (HY-10087), and A1331852 (HY-19731) were purchased from MedChemExpress. Eltanexor and selinexor were a kind gift from Karyopharm Therapeutics.

### Animal studies

All the animal experiments were in accordance with approved protocols from the Institutional Animal Care and Use Committee. The number of mice for each treatment group was between 5–10 mice to have a power of 80–90% with a standard deviation of 10%, minimizing animal use while ensuring optimal statistical power. Seven-week-old female C57BL/6 (B6.SJL-*Ptprca Pepcb/BoyJ* (CD45.1, RRID:IMSR_JAX:002014) mice were purchased from Jackson laboratory. The competitive transplant was performed on female mice aged 8–9 weeks. Mouse bone marrow cells were isolated from three *Mx1* + *Sf3b1*^*K700E*^ (CD45.2) [33] and one C57BL/6 mice (CD45.1), combined at a 4:1 ratio and injected into lethally irradiated CD45.1^+^ recipient mice. To induce recombination, the mice were treated with three doses of 1 mg/mL polyinosinic-polycytidylic acid (pIpC) (P1530, Millipore Sigma) every other day through intra-peritoneal injection one month after the transplant. After successful recombination, the mice were randomly assigned to be treated with oral gavage of vehicle control, 10 mg/kg eltanexor, 25 mg/kg BCL-family inhibitor (venetoclax, A1331852, navitoclax), or the combinations (eltanexor + venetoclax, eltanexor + A1331852, eltanexor + navitoclax) for 5 days per week for two weeks. Two weeks after finishing the treatment the experiment was ended, and bone marrow and organs were harvested. The mice were monitored for weight loss during and after treatment. Peripheral blood was collected before treatment began, each week of treatment, and at the end of the experiment. Bone marrow and organs were harvested at endpoint. Further methods can be found in the Supplementary Information.

## Results

### SF3B1 mutants are preferentially sensitive to XPO1 inhibitors

**Fig. 1:**
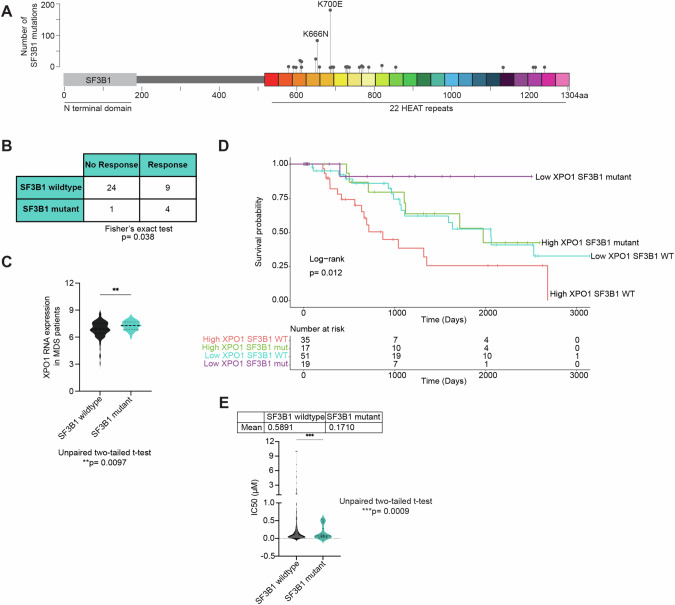
*SF3B1* hotspot mutations lead to increased sensitivity to XPO1 inhibition in myelodysplastic neoplasms (MDS). **A** Lollipop plot of the number of *SF3B1* mutations in an MDS 2020 clinical sequencing study (MSKCC, 2020) from cBioportal (*n* = 4231 samples). **B** Patients harboring *SF3B1* mutations display increased efficacy to XPO1i (selinexor and eltanexor), as shown by combining findings from a phase II clinical trial of selinexor and a phase I/II trial of eltanexor for high-risk MDS relapsed or refractory to hypomethylating agents. Data is shown as a 2 × 2 contingency table, Fisher’s exact test *p* = 0.0382. **C** XPO1 gene expression data (probe ID: 235927_at) from GSE58831 of 87 MDS *SF3B1* wildtype and 37 MDS *SF3B1* mutant patients. Data are shown as mean (*SF3B1* wildtype = 6.834, *SF3B1* mutant = 7.276), unpaired two-tailed t-test *p* = 0.0097. **D** Survival curve from GSE58831 of 185 MDS patients with high and low levels of XPO1 expression, with and without *SF3B1* mutation. Optimal cutoff for high and low XPO1 expression was determined using surv_cutpoint. Log-rank *p* = 0.012. wildtype = WT, Mutant = mut. **E** BEAT AML ex vivo drug sensitivity of SF3B1 wildtype and mutant to XPO1 inhibitor, selinexor. Unpaired two-tailed t-test *p* = 0.0009.

SF3B1 plays a key role in the spliceosome assembly and is the most commonly mutated spliceosomal gene across cancers, including MDS. The most common *SF3B1* mutations observed in MDS patients are SF3B1^K700E^ and SF3B1^K666N^ (Fig. 1A). To determine the impact of XPO1 inhibition on *SF3B1* mutations, we analyzed prior results from recent clinical trials of XPO1i, selinexor [22, 32] and eltanexor [34], in patients with high-risk MDS relapsed or refractory to hypomethylating agents (HMA). Out of 38 MDS patients, *SF3B1* was mutated in 13% of the cases (*n* = 5), and these *SF3B1* mutations were significantly associated with response to XPO1 inhibition (selinexor and eltanexor) (Fig. 1B). Additionally, in a compiled cohort of 124 MDS patients at baseline [35], *XPO1* was more highly expressed in *SF3B1* mutant MDS (Fig. 1C). This gene expression data was obtained from CD34+ bone marrow cells of MDS patients. To determine whether XPO1 expression had an impact on survival in *SF3B1* mutant patients, we analyzed the same MDS dataset [35], which showed that high XPO1 expression is significantly associated with a poor survival overall (Supplementary Fig. S1A) and specifically in the *SF3B1* mutant populations (Fig. 1D). Although these patients did have other mutations (TET2, DNMT3A, ASXL1, etc.), mutations within RNA splicing factor showed significant mutual exclusivity with minimal overlap in the downstream transcriptional effects, potentially showing distinct clinical phenotypes of RNA splicing factor mutations. Taken together, this data suggested that *SF3B1* mutations could have a targetable sensitivity to XPO1 inhibition. Given the small sample size of the SF3B1 mutant MDS patients treated in clinical trials, we further investigated the relationship between XPO1 inhibition and splicing mutations using the BEAT AML 2.0 ex vivo sensitivity data [36]. This data confirmed that *SF3B1* mutant AML was more sensitive to XPO1i. Furthermore, splicing factor mutations in *U2AF1*, but not *SRSF2*, showed increased ex vivo sensitivity when treated with selinexor (Fig. 1E; Supplementary Fig. S1B).

### Nuclear RNA retention is increased in *SF3B1* mutant cells after XPO1 inhibition

**Fig. 2:**
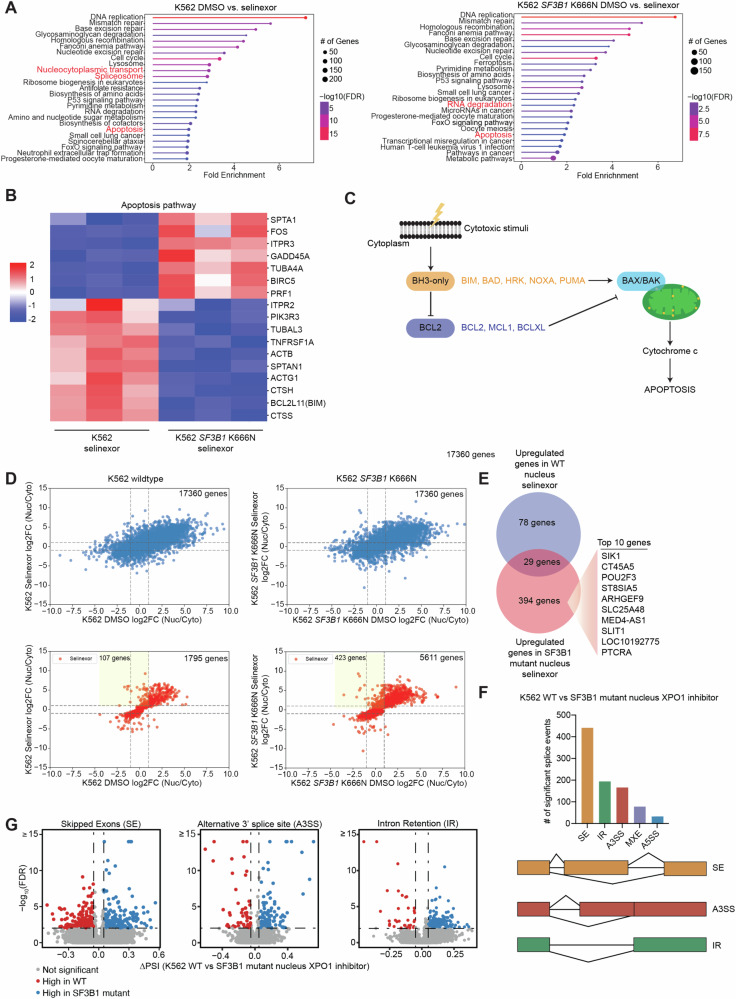
Apoptosis pathway is significantly affected with an increase in nuclear retention and alternative splicing in *SF3B1* mutant cells after XPO1 inhibition. **A** Gene Ontology (GO) term enrichment analysis of biological processes in vehicle (DMSO) vs. 200 nM selinexor (XPO1 inhibitor) treated samples for 24 h with key important pathways colored in red for *SF3B1* wildtype and mutant cells. **B** Heat map of the genes from the apoptosis pathway identified in *SF3B1* wildtype and *SF3B1* mutant cells in the presence of selinexor. **C** Schema of the apoptosis pathway illustrating the regulation of mitochondria-mediated intrinsic apoptosis pathway by BCL2 family of proteins. **D** Differentially expressed genes (DEGs) from all gene transcripts (top) and genes significantly abundant with selinexor treatment only (bottom) of *SF3B1* wildtype (left) and *SF3B1* mutant cells (right) showing increased nuclear retention in the *SF3B1* mutant cells after selinexor treatment. **E** Genes upregulated in the nucleus after XPO1 inhibitor treatment of *SF3B1* wildtype and mutant cells. **F** Identification of the significant splice events of the five major alternative splice types: skipped exons (SE), intron retention (IR), alternative’ 3’ splice site (A3SS), mutually exclusive exons (MXE) and alternative 5’ splice site (A5SS) in wildtype versus *SF3B1* mutant nucleus after XPO1 inhibition. **G** Volcano plots of SE, A3SS, and IR in wildtype versus *SF3B1* mutant nucleus after XPO1 inhibition with increased alternative splicing seen in the *SF3B1* mutant. RNA sequencing included 3 replicates per treatment group.

To study the mechanisms underlying the increased sensitivity of *SF3B1* mutants to XPO1 inhibition, global transcriptomic analysis was performed on *SF3B1* WT or K666N mutant knock-in cells exposed to vehicle (DMSO) or XPO1i (selinexor). As previously described [37, 38], XPO1 inhibition had the greatest effect on DNA repair and cell cycle-related genes in wildtype and *SF3B1*-mutant cells; however, XPO1 inhibition also affected genes related to nucleocytoplasmic transport, spliceosome, RNA degradation and apoptosis (Fig. 2A). XPO1 inhibition has been shown to induce apoptosis in various mechanisms, including, nuclear accumulation of p53 [17], suppression of survivin transcription [39], and reduction of anti-apoptotic protein MCL1 [40]. However, the underlying mechanism and how XPO1 inhibition regulates apoptotic signaling remains unclear. As such, we decided to focus on understanding the effects of XPO1 inhibition on the apoptosis pathway and the association with SF3B1 mutations. Interestingly, we noted that the response and effect of apoptosis after XPO1 inhibition was different in *SF3B1* wildtype versus *SF3B1* mutant cells at baseline and after treatment with XPO1i (Fig. 2B; Supplementary Fig. S1C). The mitochondrial, or intrinsic pathway of apoptosis is activated by cellular stress such as targeted therapy, DNA damage, chemotherapy, and radiation and is regulated by the BCL2 family of proteins [41, 42] (Fig. 2C). Upon activation, the BH3-only activator proteins (BIM and BID) bind to effector proteins (BAX and BAK), inducing apoptosis [43]. This process is inhibited by anti-apoptotic proteins such as BCL2, BCLXL, and MCL1 that can bind to activator proteins to prevent apoptosis. Pro-apoptotic sensitizer proteins (PUMA, NOXA, BAD and HRK) indirectly promote apoptosis by binding to anti-apoptotic proteins, releasing activator proteins to activate BAX/BAK [41]. Furthermore, RNA sequencing analysis revealed increase in NOXA transcription in *SF3B1* mutant cells after XPO1 inhibition (selinexor), suggesting potential increase in apoptosis in SF3B1 mutant cells (Supplementary Fig. S1D).

Given that the most enriched pathways in response to XPO1i were similar between *SF3B1* WT and mutant cells, we conducted subcellular RNA sequencing of nuclear and cytoplasmic fractions in wildtype and *SF3B1* mutant cells as we previously described [44]. We first began by looking at the global distribution in the nuclear and cytoplasmic fractions (Fig. 2D, top), followed by the differentially expressed genes after selinexor treatment (Fig. 2D, bottom). We saw an increased abundance of transcripts retained in the nucleus after XPO1 inhibition in the *SF3B1* mutant cells (Fig. 2D, E). After identifying the RNAs that selectively remain in the nucleus following XPO1 inhibition, we validated the top hits and their localization using qPCR (Supplementary Fig. S1E). Of the top hits, two RNAs identified to be differentially exported in *SF3B1* K700E mutant cells were SLC24A48 (member of potassium-dependent sodium/calcium exchanger) and SIK1 (salt inducible kinase 1). We then sought to understand how *SF3B1* mutations alter splicing globally when cells are exposed to XPO1 inhibition. We identified splicing events that were significantly different between wildtype and *SF3B1* mutant cells after XPO1 inhibition (Fig. 2F). Differential splicing was shown to be increased in the *SF3B1* mutant for skipped exons (SE), alternative 3’ splice sites (A3SS), and intron retention (IR) (Fig. 2G). We noted that with the addition of XPO1i, SF3B1 mutations showed an excerbated increase in alternative splicing. Given the changes seen in alternative splicing in the *SF3B1* mutant cells after XPO1 inhibition, we performed subcellular RNA sequencing on small RNAs. We were particularly interested in small nuclear RNAs as they are involved in the spliceosomal assembly and are exported by XPO1. Transcriptomic analysis revealed elevated small nuclear RNAs (snRNAs) in the nucleus after XPO1 inhibition for *SF3B1* mutant cells (Supplementary Fig. S1F). To explore if these changes were due to differences in small nuclear RNA abundance in the snRNA components (U1, U2, U4, U5, U6) between *SF3B1* WT and mutant cells, we performed qPCR as previously described [45] and saw no significant differences (Supplementary Fig. S1G). These results suggest that the preferential sensitivity of *SF3B1* mutants to XPO1 inhibition is due to the increased nuclear retention of select mRNAs and spliceosomal snRNAs, resulting in the perturbation of splicing and, ultimately, activation of the apoptotic pathway.

### Dynamic BH3 profiling predicts combination of BH3 mimetics and eltanexor to have increased apoptotic response

**Fig. 3:**
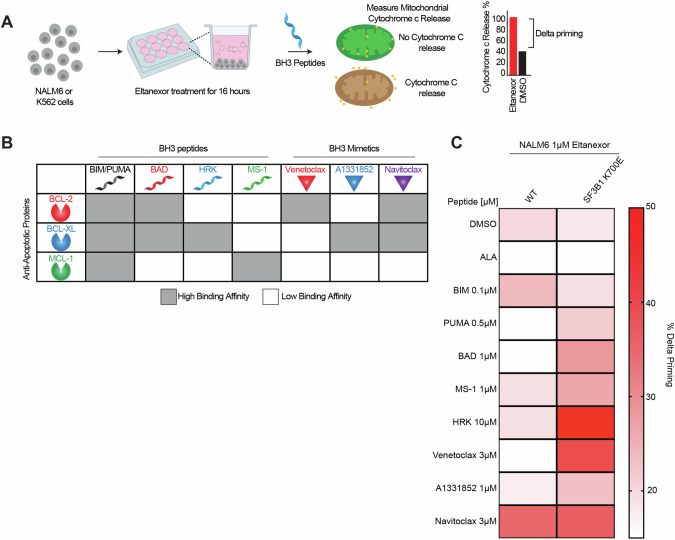
Dynamic BH3 profiling shows increased priming for BCL2 family of proteins in response to eltanexor treatment. **A** Schematic of dynamic BH3 profiling of *SF3B1* WT and mutant cells. **B** Table depicting the interaction between BH3 peptides and BH3 mimetics with anti-apoptotic BCL2 family [47]. **C** Heatmap displaying the delta priming responses of the indicated BH3 peptides in NALM6 *SF3B1* WT and mutant cells after 16 h of 1μM eltanexor treatment compared to DMSO. Delta priming is calculated as the percentage of cytochrome c loss with eltanexor treatment – percentage of cytochrome c loss with vehicle (DMSO) treatment (*n* = 3 replicates).

Given the effects on apoptosis with XPO1i in wildtype and *SF3B1* mutant cells, we next performed BH3 profiling to identify dependence on specific anti-apoptotic proteins after treatment with XPO1i (eltanexor) in wildtype and *SF3B1* mutant cells (Fig. 3A). Eltanexor has been associated with reduced brain penetration and increased tolerability and thus was used for the in vitro and in vivo testing. Dynamic BH3 profiling (DBP) is a technique that assesses drug-induced mitochondrial sensitivity to apoptosis (priming) using peptides derived from pro-apoptotic BH3-only proteins [46, 47]. Mitochondrial priming is indicated via mitochondrial outer membrane permeabilization (MOMP) measured by cytochrome c release in response to BH3 peptides. The binding patterns of BH3 peptides (BIM, BAD, MS-1, and HRK) and BH3 mimetics (venetoclax, A1331852, navitoclax) to anti-apoptotic proteins (BCL2, BCLXL, and MCL1) was used in the BH3 profiling assay [41] (Fig. 3B). The NALM6 and K562 *SF3B1* mutant cells showed increased priming to BAD (BCL2 dependence), HRK (BCLXL dependence) and PUMA peptides (Fig. 3C; Supplementary Fig. S2A–C) when treated with eltanexor. In the NALM6 cells, we saw increased priming for navitoclax, however, there was no further increase in priming of navitoclax with the *SF3B1* mutant cells. Additionally, we saw increased priming for venetoclax and A1331852 specific to the NALM6 *SF3B1* mutant cells, indicating direct mitochondrial sensitivity to BCL2 and BCLXL inhibition. The increased priming responses in the *SF3B1* mutant cells observed with HRK, surpassing that of BAD, implies a greater dependence on BCLXL rather than BCL2. The increased priming of BCLXL mimetics was also seen in K562 cells. This DBP data suggests that eltanexor increases sensitivity to BH3 mimetics that is enhanced further in the *SF3B1* mutant cells.

### CRISPR Screen reveals that loss of BCL-family of proteins sensitizes to XPO1 inhibition

**Fig. 4:**
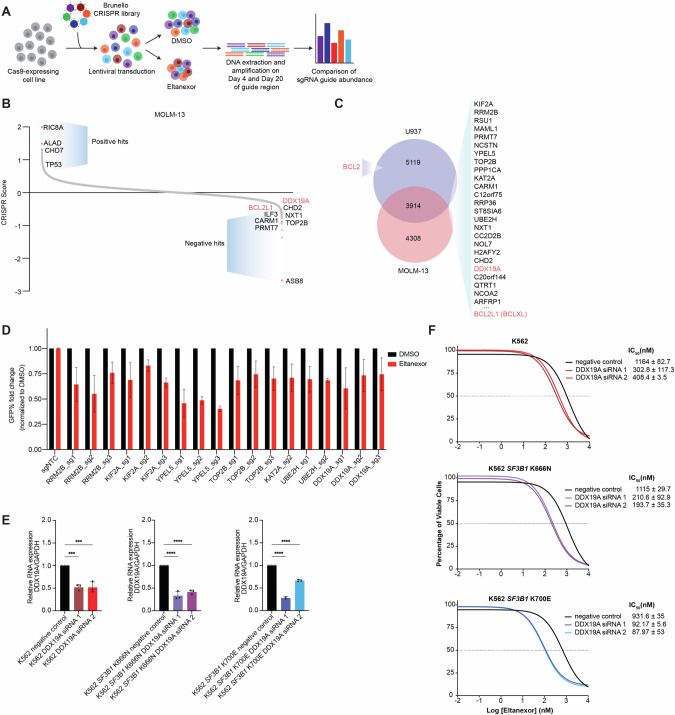
CRISPR screen identified genes that may be associated with response to XPO1 inhibition. **A** Schematic of the Brunello genome-wide CRISPR screen in AML cell line, MOLM-13 cells treated with eltanexor. **B** Genome-wide CRISPR screen of MOLM-13 cells. Colored dots indicate genes involved in BCL2 pathway. CRISPR score represents the log2 (fold-change) values of sgRNAs enriched (positive values) or depleted (negative values) after eltanexor treatment normalized to DMSO at Day 20 post-transduction. **C** Venn diagram of negative hits (sensitizers) in MOLM-13 and U937 CRISPR screens. **D** Competition-based assay of sgRNAs into MOLM-13 cells and treated with eltanexor for 48 h. Data is shown as mean + SEM. **E** RT-qPCR of DDX19A normalized to housekeeping gene GAPDH after 48 h of DDX19A siRNA-mediated gene silencing in K562 cells. **F** Dose-response curves of DDX19A siRNA knockdown cells treated with eltanexor for 48 h (*n* = 3 replicates).

In addition to the BH3 profiling, we also performed CRISPR screens to identify synthetic lethal genes that could synergize with XPO1 inhibition (eltanexor) (Fig. 4A). Here we performed a genome-wide Clustered Regularly Interspaced Short Palindromic Repeats (CRISPR) screen consisting of 77,441 single guide RNAs (sgRNAs) targeting 19,115 genes in MOLM-13 and U937 cells with and without eltanexor [48] (Fig. 4B; Supplementary Fig. S3A). We analyzed for changes in abundance at day 20 post-transduction by measuring the average fold change (eltanexor/DMSO) of all sgRNAs targeting a given gene, and top-scoring candidates were classified as genes that sensitize (negative CRISPR score) or confer resistance (positive CRISPR score) to XPO1 inhibition. Our screens identified previously characterized genes, including TP53, whose loss has been shown to confer resistance [40, 49–51] as well as ASB8 which sensitizes to XPO1 inhibition [52]. We found 3914 genes to sensitize to XPO1 inhibition in both CRISPR screens (Fig. 4C). Of note, BCL2L1 (BCLXL) knockout was identified as sensitizing in both screens (MOLM13 and U937) and BCL2 was identified only in U937 screen. As predicted from the BH3 profiling, we observed that loss of BCL2 and BCLXL sensitized *SF3B1* mutant cells to eltanexor. Additionally, we identified the DEAD-box helicase DDX19A, a gene involved in nucleocytoplasmic transport, as a top sensitizer in both screens. We then further validated the top synthetic lethal genes using competition-based assays and confirmed gene knockout (KO) candidates conferred sensitivity to eltanexor treatment (Fig. 4D; Supplementary Fig. S3B). Given the role of DDX19A in nuclear export, we further validated the relationship between loss of DDX19A and sensitivity to XPO1 inhibition. We performed siRNA knockdown of DDX19A and identified that reduction of DDX19A led to sensitivity to eltanexor that was further enhanced in *SF3B1* mutant cells (Fig. 4E, F).

### *SF3B1* mutant cells show increased synergy and apoptosis with eltanexor and venetoclax

**Fig. 5:**
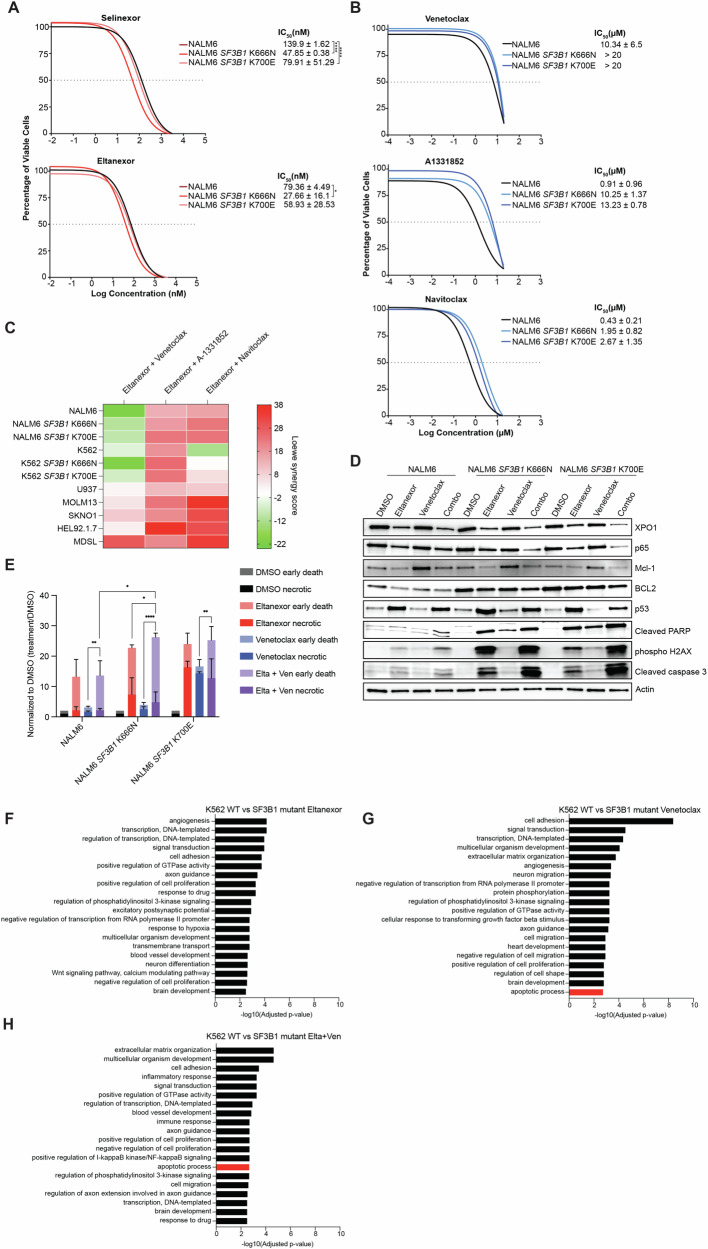
*SF3B1* mutant cells show increased synergy with the combination of XPO1 inhibitor and BCL inhibitors. **A** Dose-response curves of NALM6 cells treated with XPO1 inhibitors, eltanexor and selinexor, for 72 h (*n* = 4 replicates). Two-way ANOVA. **B** Dose-response curves of NALM6 cells treated with BCL inhibitors, venetoclax, A-1331852, and navitoclax, for 72 h (*n* = 4 replicates). **C** Heat map of the combination of eltanexor and BCL inhibitors in *SF3B1* WT and mutant cells and other AML cell lines using Loewe on SynergyFinder. >10 are synergistic, <−10 are antagonistic. (*n* = 3 replicates). **D** Western blot analysis of XPO1 and BCL2 targets after treatment with vehicle, 200 nM eltanexor, 1 μM venetoclax, and combination of 200 nM eltanexor and 1μM venetoclax for 24 h. **E** Stacked bar plots of Annexin V and propidium iodide staining showing the early death and necrotic cells of NALM6 isogenic cells after 72 h of treatment with eltanexor, venetoclax, and the combination (*n* = 3 replicates) normalized to DMSO. Two-way ANOVA, statistics shown are for early death comparisons. **F** GO pathway enrichment analysis of the differentially expressed genes between wildtype and SF3B1 mutant with eltanexor treatment. **G** GO pathway enrichment analysis of the differentially expressed genes between wildtype and *SF3B1* mutant with the venetoclax treatment. **H** GO pathway enrichment analysis of the differentially expressed genes between wildtype and *SF3B1* mutant with eltanexor with venetoclax treatment. RNA sequencing included 3 replicates per treatment group.

Following the identification of BCL2 and BCLXL as genetic targets that sensitized to XPO1 inhibition in the CRISPR screen and have increased delta priming in the BH3 profiling, we tested the combination of eltanexor with selective BCL2, BCLXL, or BCL2/BCLXL inhibitors in vitro and in vivo. We first measured the half-maximal inhibitory concentration (IC50) of wildtype and *SF3B1* mutant cell lines when exposed to first and second-generation XPO1 inhibitors, selinexor and eltanexor, respectively (Fig. 5A; Supplementary Fig. S4A). We found increased sensitivity in the *SF3B1* mutant cell lines towards the XPO1 inhibitor in comparison to wildtype cells. We also performed IC50s of venetoclax (BCL2 inhibitor), A1331852 (BCLXL inhibitor), and navitoclax (BCL2 and BCLXL inhibitor) in *SF3B1* wildtype and mutant cells and did not observe increased sensitivity in the *SF3B1* mutant cells (Fig. 5B; Supplementary Fig. S4B). We then tested the combination of XPO1i (eltanexor) with venetoclax, A1331852, and navitoclax using increasing concentrations of each drug in *SF3B1* wildtype, *SF3B1* mutant, and various AML/MDS cell lines (Fig. 5C). Using the Loewe model to calculate synergy, we found that *SF3B1* mutant cells had increased synergy, particularly with the combination of eltanexor and A1331852 as well as with eltanexor and navitoclax. Western blot analysis of the combination of eltanexor with A1331852 and navitoclax showed decreased levels of MCL1 with the combination, however, no significant reduction in p53 (Supplementary Fig. S4C). Interestingly, western blot analysis of the combination of eltanexor and venetoclax revealed upregulation of MCL1 with venetoclax treatment that was decreased with the combination of eltanexor and venetoclax (Fig. 5D; Supplementary Fig. S4D). Consistent with the greater effects in the SF3B1 mutant cells, expression of DNA damage and apoptosis markers (cleaved caspase 3, phospho-H2AX, and cleaved PARP) were found to be increased specifically in the *SF3B1* mutant cells. To confirm the type of cell death, we performed an apoptosis detection assay and saw a significant increase in early cell death in *SF3B1* mutants with the eltanexor and venetoclax combination (Fig. 5E). To characterize the global transcriptomic effect of the combinations in the *SF3B1* wildtype and *SF3B1* mutant cells, we performed RNA sequencing on the single agent-treated cells (DMSO, eltanexor, venetoclax, A1331852, navitoclax) and on the combination treated cells (eltanexor + venetoclax, eltanexor + A1331852, and eltanexor + navitoclax). Principal component analysis of the RNA-sequencing data revealed that *SFB31* mutant cells clustered distinctly from *SF3B1* wildtype cells (Supplementary Fig. S4E). Cells treated with the addition of eltanexor were clustered separately from cells treated with single agent BCL-family inhibitor. We then examined differentially expressed pathways by comparing *SF3B1* mutant cells to wildtype cells for each treatment group. There was no increase in apoptosis after eltanexor treatment specific to the SF3B1 mutant cells (Fig. 5F). We found that A1331852 and navitoclax significantly increased the expression of apoptosis pathway genes in the *SF3B1* mutant cells, but this increase was less profound with the addition of XPO1i (Supplementary Fig. S4F, G). Conversely, with venetoclax there was an increase in the significance of representation of the apoptosis pathway genes that was further enhanced with the combination of eltanexor and venetoclax (Fig. 5G, H). Altogether the in vitro drug sensitivity data, the CRISPR screen and BH3 profiling of *SF3B1* mutant cells revealed that the combination of the XPO1i eltanexor with the BCL2 inhibitor venetoclax demonstrated an increase in apoptotic pathway, sensitivity to XPO1i, and synergistic cell killing.

### The combination of eltanexor and venetoclax selectively targets Sf3b1 mutant cells in vivo

**Fig. 6:**
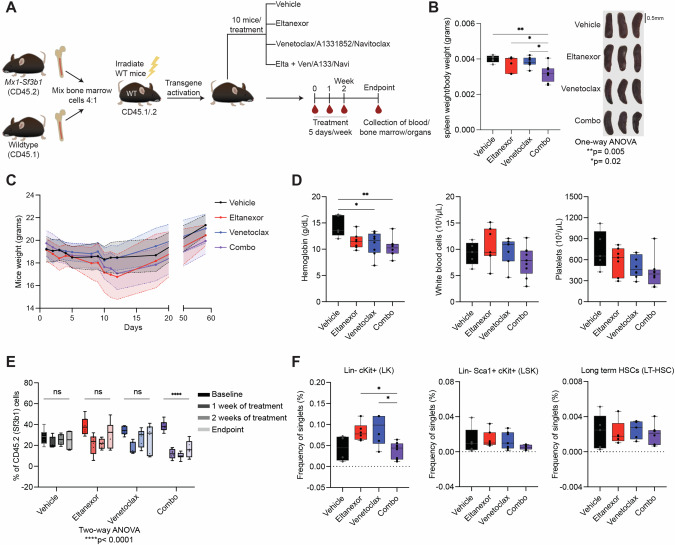
Combination of XPO1 inhibitor and BCL2 inhibitor, venetoclax, led to a decrease of Sf3b1 mutant cells. **A** Schema for the transgenic mice treatment showing Sf3b1 mutant (CD45.2) and wildtype (CD45.1) bone marrow cells combined at a 4:1 ratio and injected into irradiated wildtype mice. Following transgene activation, mice were treated with vehicle, eltanexor (10 mg/kg), BCL inhibitor (25 mg/kg), and the combination of eltanexor and BCL family inhibitor for two weeks. Collection of blood occurred at baseline, after a week of treatment, after second week of treatment, and at endpoint. **B** Bar chart of reduced size and weight of spleen in combination treated mice with representative images of the spleens. Scale bar: 0.5 mm. One-way ANOVA **p = 0.005, *p = 0.02. **C** Comparison of the body weight between the treatment groups of vehicle, eltanexor, venetoclax, and the combination of eltanexor and venetoclax. Data are shown as mean ± standard deviation. **D** Effect of eltanexor, venetoclax, and the combination on hematological parameters (hemoglobin, white blood cells, and platelets) at the endpoint of experiments. Data are shown as mean ± standard deviation, one-way ANOVA **p = 0.005, *p = 0.02. **E** Violin plot of CD45.2+ compartment (Sf3b1 mutant cells) in peripheral blood before treatment, after one week of treatment, after two weeks of treatment, and at endpoint. Data are shown as mean ± standard deviation, two-way ANOVA ****p < 0.0001. **F** Percentage of Lin^−^cKit^+^ (LK), Lin^−^Sca1^+^cKit^+^ (LSK), and long-term hematopoietic stem cells (LTHSC) in CD45.2+ compartment, one-way ANOVA. Elta = eltanexor, Ven = venetoclax, A133 = A1331852, Navi = navitoclax, Combo = eltanexor and venetoclax.

The heterozygous Sf3b1^K700E^ conditional knock-in mice has previously been shown to develop macrocytic anemia due to impaired erythropoiesis, myelodysplasia, and aberrant splicing, reflecting the phenotype of the SF3B1 mutation seen in MDS patients [33]. As such, we subsequently tested the therapeutic efficacy of the combinations of XPO1i with BCL inhibitors in vivo using Sf3b1^K700E^ conditional knock-in mice competitive transplant assays (Fig. 6A). Considering that the Sf3b1^K700E^ hematopoietic stem and progenitor cells (HSPCs) have a competitive disadvantage in bone marrow transplantation assays, we transplanted the bone marrow of Sf3b1^K700E^ (CD45.2) and wildtype (CD45.1) mice at a 4:1 ratio into wildtype irradiated mice (CD45.1). The mice were then treated with vehicle, 10 mg/kg eltanexor, 25 mg/kg single agent BCL-family inhibitor, and the combination of eltanexor with a BCL-family inhibitor. Given the results we saw in vitro, we were most interested to test the combination of eltanexor and venetoclax. We saw a decrease in spleen weight with the combination (Fig. 6B). However, there was not a significant decrease in mice weight or hematological parameters that was specific or as a consequence of the combination (Fig. 6C, D; Supplementary Fig. S5A). With the treatment of eltanexor alone and venetoclax alone there was a decrease in the *Sf3b1*-mutant burden marked by the CD45.2 compartment, a decrement that recovered to baseline levels over time. Interestingly, with the combination of eltanexor and venetoclax there was a significant decrease in the *Sf3b1* mutant cells in the peripheral blood that was maintained (Fig. 6E). We also saw a decrease in CD3+ and CD11b+CD45.2 cells with the combination of eltanexor and venetoclax (Supplementary Fig. S5B). We used flow cytometry of the bone marrow hematopoietic progenitor and stem cell compartments (Supplementary Fig. S5C) to analyze LSKs (Lin^-^Sca1^+^cKit^+^), LKs (Lin^−^cKit^+^), multipotent progenitors (MPP), long-term HSCs (LT-HSC), short-term HSCs (ST-HSC), common myeloid progenitors (CMP), megakaryocytic-erythroid progenitors (MEP), and granulocyte-monocyte progenitors (GMP). In LK, LSK, LT-HSC, MPP, and CMP compartments, there was a trend towards decrease in *Sf3b1* mutant cells with the combination of eltanexor and venetoclax (Fig. 6F; Supplementary Fig. S5D, E). Specifically, the LKs showed a significant decrease in Sf3b1 mutant cells with the combination (Fig. 6G). To further emphasize the mutant-selective killing, we performed a colony-forming unit (CFU) assay using bone marrow from Vav-Cre Sf3b1^WT^ control and Vav-Cre Sf3b1^K700E^ mice (Supplementary Fig. S5F). In this assay, we observed a significant reduction in the progenitor colonies in the Sf3b1^K700E^ mice treated with the combination of eltanexor and venetoclax in comparison to single-agent and the control mice, suggesting the combination has a preferentially selective effect on SF3B1 mutant cells. We also tested the combination of eltanexor and A1331852 as well as the combination of eltanexor and navitoclax. Spleen weight decreased with the combination of eltanexor and A1331852 and increased with the combination of eltanexor and navitoclax (Supplementary Fig. S6A). With both combinations, there was slight toxicity seen with a decrease in mice weight and hematological parameters that was specific to the combination treatment (Supplementary Fig. S6B, C). With the combination of eltanexor and A1331852 there was a significant decrease in CD45.2 cells in the peripheral blood, similar to the decrease observed with the combination of eltanexor and venetoclax (Supplementary Fig. S6D). However, with eltanexor and navitoclax there was no significant change in the CD45.2 cells, suggesting that *SF3B1* mutant cells may not have a specific sensitivity to this combination. We then examined the bone marrow compartments and saw trends toward decrease in LSK, LK, LT-HSC, and ST-HSC with eltanexor and A1331852 (Supplementary Fig. S6E). Although we observed decreases in peripheral blood and bone marrow in the *SF3B1* mutant cells with the combination of eltanexor and A1331852, given the weight loss and toxicity seen in these mice, the combination of eltanexor and venetoclax appeared to be a more promising combination for future development in clinical trials for patients with *SF3B1* mutant MDS.

## Discussion

XPO1 is involved in the export of many critical cellular regulators, making XPO1 inhibition a broadly relevant therapeutic strategy, yet the exact mechanisms of action of XPO1 inhibition may be unique to different cancer subtypes and specific mutations. Here, we found *SF3B1* mutation to be significantly associated with XPO1 expression and response to XPO1 inhibition in clinical trials of two XPO1 inhibitors, selinexor and eltanexor, in MDS. Furthermore, XPO1 expression was associated with worse survival in MDS*. SF3B1* is the most commonly mutated spliceosomal gene in MDS and, although *SF3B1* mutations are typically associated with good prognosis [53, 54], we saw a decrease in survival with increased XPO1 expression in *SF3B1* mutations. Furthermore, *SF3B1* and *U2AF1* mutant, but not *SRSF2* mutant AML cells from the BEAT AML ex vivo drug screening showed preferential sensitivity to XPO1 inhibition. Thus, a subset of *SF3B1* mutant MDS/AML patients have poorer outcomes and XPO1 represents a potential therapeutic target in this population. This could potentially extend to other splicing mutant myeloid neoplasms although more specific studies are needed.

Despite the significant role *SF3B1* mutations play in the development and progression of MDS, targeted therapies against the spliceosome have failed to result in approved therapeutics thus far. The importance of RNA export in the spliceosome and how XPO1 inhibition affects this process had not previously been explored. In this study, we analyzed the effects of XPO1 inhibition on RNA export to better understand the mechanism behind the sensitivity of XPO1 inhibition in *SF3B1*-mutant MDS and AML. We found that XPO1 inhibition leads to increased nuclear retention of small nuclear RNAs and select messenger RNAs leading to increased alternative splicing, an effect that is further amplified in the SF3B1 mutant cells.

The second-generation XPO1 inhibitor, eltanexor, appears to have increased efficacy and tolerability in a small phase 2 study of MDS [32, 34]; however, despite the promising efficacy of XPO1 inhibition in *SF3B1*-mutated MDS, maximizing efficacy and minimizing toxicity remains a challenge, which forms the rationale for investigating combination therapies. Given this, we performed a genome wide CRISPR screen that identified targets synergistic with XPO1 inhibition. BCLXL was identified in two screens and BCL2 was identified in one of the screens. BCL2 is often found to be overexpressed in hematological malignancies and we found RNAs involved in the apoptotic pathway to be retained in the nucleus after XPO1 inhibition; thus, the combination of a BCL-family inhibitor and XPO1 inhibitor seemed to be a promising opportunity to further enhance programmed cell death. As such, we tested out the combinations of eltanexor and venetoclax (BCL2 inhibitor), eltanexor and A1331852 (BCLXL inhibitor), and eltanexor and navitoclax (BCL2/BCLXL inhibitor). Our BH3 profiling results indicated increased priming in the *SF3B1* mutant with venetoclax and navitoclax, which was confirmed in our in vitro studies that showed increased apoptosis, specifically in the *SF3B1* mutant cells. Furthermore, we tested out the combinations in vivo and although eltanexor and A1331852 showed a decrease in Sf3b1 mutant cells in the peripheral blood and bone marrow, weight loss and toxicity were observed in these mice in excess of the venetoclax combination treated mice.

Although the combination of eltanexor and BCLXL inhibition demonstrated the strongest synergistic effect, the associated toxicity seen in vivo poses challenges for clinical application; however, if newer BCLXL inhibitors or degraders are shown to have improved safety profiles this combination could be revisited. Thus, because venetoclax is currently clinically available for use in MDS/AML, we suggest the combination of eltanexor and venetoclax should warrant further exploration in the context of clinical trials. Recent human data has also shown that venetoclax can overcome the poor prognosis of spliceosomal mutant AML patients [55]. Additionally, it has been previously reported that the combination of an XPO1 inhibitor with venetoclax showed a synergistic response in AML patient samples that were refractory to venetoclax in clinical settings [56]. Our study has identified the first genotype-specific enhanced effect of XPO1 inhibition with mechanistic support for the combination of eltanexor and venetoclax as a promising targeted strategy for therapy for SF3B1-mutant MDS and AML, addressing a significant lack of approved therapies targeting spliceosomal mutations.

### Supplementary information

Supplemental Materials

## Data Availability

All relevant data is included in the manuscript and Supplementary Information. RNA sequencing data has been uploaded to GEO under Accession ID GSE255179. Any additional data or code that support the findings of this study are available from the corresponding author upon reasonable request.
