# Supplementary material for: Altered RNA export by SF3B1 mutants confers sensitivity to nuclear export inhibition
Source: Leukemia. 2024 Jul 13;38(9):1894–905. doi: 10.1038/s41375-024-02328-1 (PMC11347370; doi:10.1038/s41375-024-02328-1)
Supplement: Supplementary file 1 — Supplemental Materials [file 41375_2024_2328_MOESM1_ESM.pdf]

<u>SUPPLEMENTARY MATERIALS</u>

A) Supplementary Methods……………………………………………………….2-6

B) Supplementary Figures with Legends…………………………………….7-15

A) Supplementary Methods

Flow cytometry analysis

Single cell suspensions were prepared from the transplant mice bone marrow or peripheral blood. Red blood cells were lysed using ammonium chloride potassium (ACK) lysis buffer (A1049201, Gibco) and resuspended into cold phosphate-buffered saline (PBS) (20012-027, Gibco) with 2% bovine serum albumin (BSA) (A9647, Sigma Aldrich). Total cells were quantified using the Vi-CELL BLU Cell Viability Analyzer and $3x10^7$ cells were blocked by incubation with Brilliant Stain Buffer (563794, BD Biosciences) for 30 minutes. The cells were then incubated with antibodies for 30 minutes on ice. For the peripheral blood, cells were stained with the following APC B220 (103212, RRID:AB_312997), FITC CD3 (100204, RRID:AB_312660), APC/Cyanine 7 Gr-1 (108424, RRID:AB_2137485), PE CD11b (101208, RRID:AB_312791), PerCP/Cyanine 5.5 CD45.2 (109828, RRID:AB_893350), CD45.1 Alexa Fluor 700 (110724, RRID:AB_493733), and DAPI at a 1:1000 ratio. For hematopoietic stem and progenitor cell analysis, cells were stained with the following APC/Cyanine 7 lineage antibodies from Biolegend CD11b (101226, RRID:AB_830642), B220 (103224, RRID:AB_313006), CD4 (100526, RRID:AB_312727), CD8a (100714, RRID:AB_312753), Gr-1 (108424, RRID:AB_2137485), Ter119 (116223, RRID:AB_2137788). Cells were also stained with antibodies against AF700 CD45.1 (110724, RRID:AB_493733), FITC CD45.2 (109806, RRID:AB_313443), Brilliant Violet 650 Sca-1 (108143, RRID:AB_2629684), Brilliant Violet 785 c-Kit (105841, RRID:AB_2629799), APC CD34 (128612, RRID:AB_10553896), PE CD16/32 (156606, RRID:AB_2800704), Brilliant Violet 421 CD150 (115943, RRID:AB_2562190), PE/Cyanine 7 CD48 (103424, RRID:AB_2075049), and red fluorescent live dead reactive dye (L34971A, Invitrogen). FACS analysis was performed on a CytoFlex LX Flow Cytometer and data analysis was performed using FlowJo software (RRID:SCR_008520).

Cell viability assays

Cell viability assays were done as previously described (55). Cell lines were seeded at 10,000 cells/well and treated with compound (20µM-5nM, 0.1% DMSO) in white 96-well plates for 72 hours with a minimum of four technical replicates per concentration. Viability was quantified using CellTiter-Glo 2.0 (Promega) according to manufacturer's instructions. Luminescence values were normalized to the DMSO vehicle treated well. IC_{50} values were interpolated from non-linear fit of log (inhibitor) versus response using GraphPad Prism (RRID:SCR_002798). To assess the efficacy of the combination of XPO1i compounds and BCL inhibitors, cells were seeded in a 6 x 6 matrix of increasing doses of each compound. Synergy was analyzed using the Loewe model on the SynergyFinder software (synergy.fimm.fi, RRID:SCR_019318). A synergy score above 10 indicates synergistic drug effects and values below -10 indicates antagonistic drug effects. Experiments were performed in at least duplicate or triplicate.

Western blot

Cell lines were treated with the dose indicated for 24 hours. Protein lysates were prepared using the Pierce IP Lysis buffer (87788, ThermoFisher) with protease and phosphatase inhibitor (78446, ThermoFisher), rotated for 20 minutes, centrifuged at the highest speed for 15 minutes at 4°C. Protein concentration was determined using the Pierce BCA Protein Assay kit (23227, ThermoFisher). Fifteen micrograms of protein were loaded per lane and loaded onto 4-12% bis-

tris protein gels. PVDF membranes were probed with the following primary antibodies: β-actin (A1978, Millipore Sigma, RRID:AB_476692), XPO1 (sc-74454, Santa Cruz, RRID:AB_1122704), p53 (sc-126, Santa Cruz, RRID:AB_628082), p65 (8242, Cell Signaling, RRID:AB_10859369), BCL2 (15071, Cell Signaling, RRID:AB_2744528), MCL1 (39224, Cell Signaling, RRID:AB_2799149), BCLXL (2764, Cell Signaling, RRID:AB_2228008), cleaved caspase 3 (9664, Cell Signaling, RRID:AB_2070042), phospho-H2AX (05-636, Millipore Sigma, RRID:AB_309864), and cleaved PARP (5625, Cell Signaling, RRID:AB_10699459) overnight. Blots were then incubated with HRP-conjugated secondary Mouse and Rabbit (7076 and 7074, Cell Signaling, RRID:AB_330924 and RRID:AB_2099233) antibodies. Following incubation, blots were developed with ECL Detection reagent (170-5061, BioRad) or SuperSignal™ West Femto Maximum Sensitivity Substrate (34096, Thermo Fisher). Densitometry was performed using ImageJ (RRID:SCR_003070).

Dynamic BH3 profiling

DynamicBH3 profiling was performed as described previously (56). Briefly, this involves the cell lines (NALM6 and K562) to be exposed to eltanexor or vehicle (DMSO) for 16 hours followed by BH3 profiling involving BH3 peptides and mimetics. The delta priming is calculated as cytochrome c loss in eltanexor- cytochrome c loss in vehicle.

Genome-wide CRISPR Screen

CRISPR screens were performed as previously described (57). MOLM-13 and U937 Cas9-expressing cells were transduced with whole genome Brunello sgRNA library at a low multiplicity of infection (~0.3) to obtain 500 cells per sgRNA. Genomic DNA was harvested on day 4 and day 20 post-transduction using the QIAamp DNA mini kit (51306, QIAGEN). The CRISPR score were calculated by normalized using (sgRNA read count/total read count) x 100,000. Following this, $\log_2$ fold change was calculated using normalized read count eltanexor/normalized read count DMSO.

Knockdown studies

Cells were transfected with Silencer Select siRNAs targeting DDX19A (AM16708 and 4392420, Thermo Fisher) using Lipofectamine 3000 transfection reagent (L3000, Invitrogen). Silencer Select Negative Control No. 1 siRNA (A390843, Thermo Fisher) was used as a negative control. Cells were transfected for 48 hours after which RT-qPCR and cell viability assays were performed.

RT-qPCR Gene Expression analysis

Total RNA was extracted from whole cells using the RNEasy mini kit (74106, QIAGEN) according to the manufacturer's instructions. RNA quality and quantity was measured using the Nanodrop spectrophotometer. Total RNA (500ng) was reverse-transcribed into double stranded cDNA using SuperScript III First-Strand Synthesis System (18080051, Invitrogen). Quantitative RT-PCR amplifications were performed using the following Taqman Gene Expression probes: GAPDH (Hs02758991_g1), XPO1 (Hs_00185645_m1), DDX19A (Hs01035515_m1), BCL2L1 (Hs00236329_m1), SIK1 (Hs00545020_m1), SLC25A48 (Hs00415075_m1), and CT45A2 (Hs04190919_mH). The expression level of the target genes was calculated using the Delta-Delta Ct method. GAPDH was used as the internal control.

Apoptosis analysis
Cells were stained with FITC-conjugated Annexin V in Annexin V-binding buffer and propidium iodide according to manufacturer's instructions (556547, BD Biosciences). Data was acquired on a CytoFlex LX Flow Cytometer and data analysis was performed using FlowJo software.

Colony forming assay
Total mouse bone marrow cells were plated at 2,000 cells per well in triplicates with methylcellulose media (M3434, StemCell Technologies) supplemented with 1% P/S. Cells were treated with vehicle, eltanexor (200nM), venetoclax (1μM), or the combination. After 14 days, the colonies were counted.

Immunoprecipitation
30 million cells were treated with either vehicle (DMSO) or 200nM of eltanexor for 24 hours at 37°C. Cells were harvested and washed with ice cold 1X PBS. Cells were lysed in IP lysis buffer containing protease and phosphatase inhibitors. Cell extracts were incubated overnight at 4°C with 50μl of protein-A Dynabeads crosslinked with anti-BIM antibody (2819, Cell Signaling, RRID:AB_10692515). Proteins were then eluted with glycine HCl and 2X Laemelli sample buffer and boiled for 10 minutes at 70°C. Supernatant was taken, and western blot analysis was performed.

Nuclear and cytoplasmic RNA extraction and messenger RNA sequencing
K562 wildtype and *SF3B1* mutant cells were treated with 200nM selinexor and without selinexor for 24 hours with three replicates each. To separate nuclear and cytoplasmic fractions, pellet the cells via centrifugation for 5 minutes at 1500RPM and discard the supernatant. The pellet was then resuspended into 300μL of ice cold 0.25x lysis buffer (Tris-Cl pH 8.0, NaCl, $MgCl_2$, Nonidet P-40). The pellet was kept on ice for two minutes and rotate the tube every 45 seconds to ensure proper lysis followed by centrifugation at highest speed at 4°C for two minutes. After centrifugation, the supernatant was carefully removed and placed in a new tube as this was the cytoplasmic fraction. For the remaining nuclear fraction pellet, 500μL of ice-cold PBS was added and centrifuged at 1500RPM for 5 minutes. The QIAGEN RNeasy mini kit was then used for purified RNA. To confirm the separation of the nuclear and cytoplasmic fractions, RT-qPCR was performed as described above using MALAT1 (Hs00273907_s1) as the nuclear marker and TUG1 (Hs00215501_m1) as the cytoplasmic marker. To identify the mRNAs that are differentially exported by XPO1, ribosomal RNA depletion method with the TruSeq stranded Total RNA library was used with 50 million paired end reads.

RNA sequencing data analysis and splicing quantification
For RNA-seq data analysis (Total and polyA fraction), FASTQ data were processed with Trimmomatic (v0.32, RRID:SCR_011848) (58) to remove low-quality and short reads and then aligned to the human genome hg19 using STAR aligner (v2.7.9a, RRID:SCR_004463) (59) with default parameters. RSEM (v1.3.1) (60) was used to obtain expected gene counts against the human gene reference Gencode (v41, RRID:SCR_014966) or RefSeq for subcellular analysis (polyA). Differential expression was determined using DESeq2 (61) and R (v4.2.0). A gene was considered detected if the TPM (Transcripts Per Million) >1 in at least two replicates of one

sample and significantly changed if the adjusted p-value was < 0.05 and fold change >1.5 and <−1.5.

For alternative splicing (AS) events detection, reads were mapped to the human genome hg19 using STAR aligner (v2.7.9a). The Selinexor replicate bam files and their control (DMSO) replicate bam files with the Gencode (v41) annotation file were used as input for rMATS (v4.1.2) (62), to report five types of the differential AS events. SE (skipped exon), MXE (mutually exclusive exons), A3SS (alternative 3′ splice site), A5SS (alternative 5′ splice site) and RI (retained intron). Events with |inclusion level difference| > 0.05, P < 0.05 and FDR < 0.05 were identified as significantly differentially expressed AS events. Custom R code using the ggplot graph library was used to generate additional volcanos plots from the analysis results.

The gene ontology (GO) analysis were performed with ShinyGO (RRID:SCR_019213) (63) using the differentially expressed genes or differentially alternative splicing genes.

Small RNA extraction and sequencing
A similar nuclear cytoplasmic fractionation method as described above was performed but instead with the miRNeasy Tissue/Cells Advanced Micro Kit (217684, QIAGEN) to identify differentially exported small nuclear RNAs. The small RNA sequencing was done with a size selection step with the Perkin Elmer Next Flex Small RNA library preparation kit on the Illumina sequencing platform with 50 million single end reads.

Small RNA sequencing data analysis
Data analysis was performed as described in (64). Briefly, sequencing reads were trimmed with Trimmomatic (v0.32) (58) to remove low-quality and short reads (shorter than 17 bp). Reads were aligned against human elements in RepBase (v23.08) with STAR (v2.7.9a) (59), and the unmapped output was then mapped against the human genome (hg19), allowing three mismatches and keeping all uniquely aligned reads.

Combination treatment mRNA sequencing
K562 wildtype and SF3B1 K666N cells were treated with either vehicle (DMSO), 200nM eltanexor, 1µM BCL-family inhibitor (venetoclax, A1331852, or navitoclax) and the combinations (eltanexor + venetoclax, eltanexor + A1331852, and eltanexor + navitoclax) for 24 hours with two technical replicates. After 24 hours, RNA extraction was done using the QIAGEN RNeasy mini kit. RNA library preparation was done with poly(A) selection on the Illumina HiSeq 2x150bp. The sequence reads were trimmed using Trimmomatic and mapped using GRCh38 to STAR aligner. Feature counts were calculated using subhead package v.1.5.2. DESeq2 was used for differential expression analysis with genes with adjusted p-value < 0.05 and log2 fold change > 1 were considered differentially expressed genes.

Statistical analysis
Statistical analyses for these experimental results was performed using GraphPad Prism and R. Specific statistical analysis details are located in the figure legends. Data were analyzed by unpaired two-tailed Student's t-test or analysis of variance (ANOVA) for more than two groups comparison. For unequal distribution, Welch's t-test was performed where appropriate. Kaplan-Meier survival curve used the log-rank statistics in R, with p <0.05 considered significant. Error

bars represent the standard error of the mean (SEM) and the data is shown as significant when $p < 0.05$ with a 95% confidence interval. All experiments are reported as at least mean triplicate ± SEM unless otherwise indicated in the figure legend. Investigators were not blinded to any of the experiments and the outcome assessment.

B) Supplementary Figures with Legends

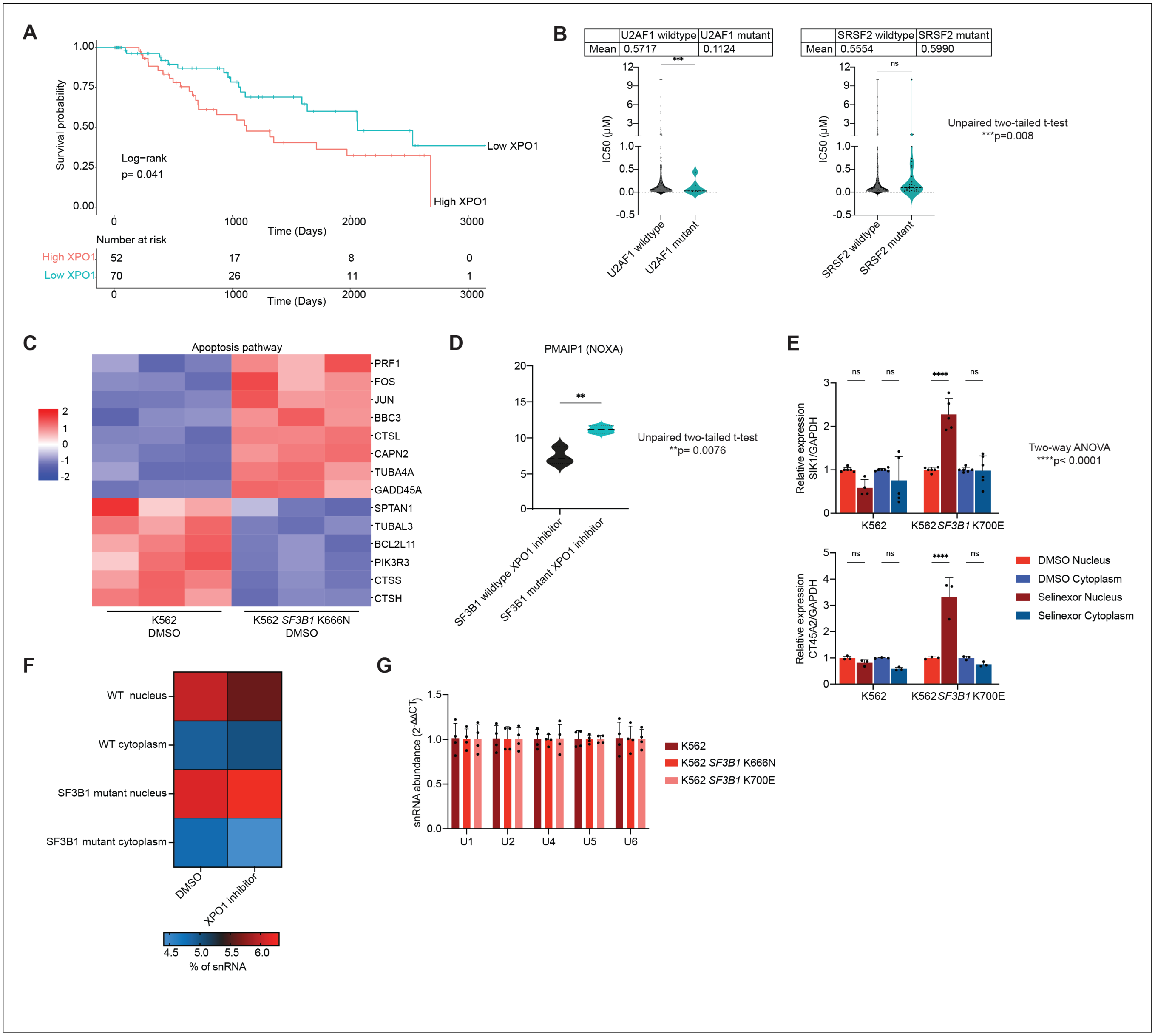

Supplementary Figure S1. ***SF3B1*** **mutations have increased small nuclear RNAs in the nucleus after XPO1 inhibition.** (**A**) Survival curve from GSE58831 of 185 MDS patients with high and low levels of XPO1 expression. Optimal cutoff for high and low XPO1 expression was determined using surv_cutpoint. Log-rank p=0.041. (**B**) BEAT AML ex vivo drug sensitivity of splicing mutation bearing patient samples, *U2AF1* or *SRSF2*, to the XPO1 inhibitor, selinexor. Unpaired two-tailed t-test. (**C**) Heat map of the genes from the apoptosis pathway identified in SF3B1 wildtype and SF3B1 mutant cells before treatment. (**D**) RNA sequencing analysis

showing significantly increased levels of NOXA in the SF3B1 mutant after XPO1 inhibition. Data is shown as the mean unpaired two-tailed t-test (n=3 replicates, NOXA p=0.0076). (**E**) Validation via RT-qPCR of the upregulation of genes, SIK1 and SLC24A48, in the nucleus of SF3B1 mutant cells after XPO1 inhibition (n ≥ 3 replicates). Data is mean ± standard deviation, two-way ANOVA p<0.0001. (**F**) Heat map of small nuclear RNAs before and after XPO1 inhibition shown as the average of 3 independent biological replicates. (**G**) Expression levels of each small nuclear RNAs (U1, U2, U3, U4, U5, and U6) in K562 cells relative to control primers (5S rRNA, RN7SL1, and RN7SK) (n=4 replicates).

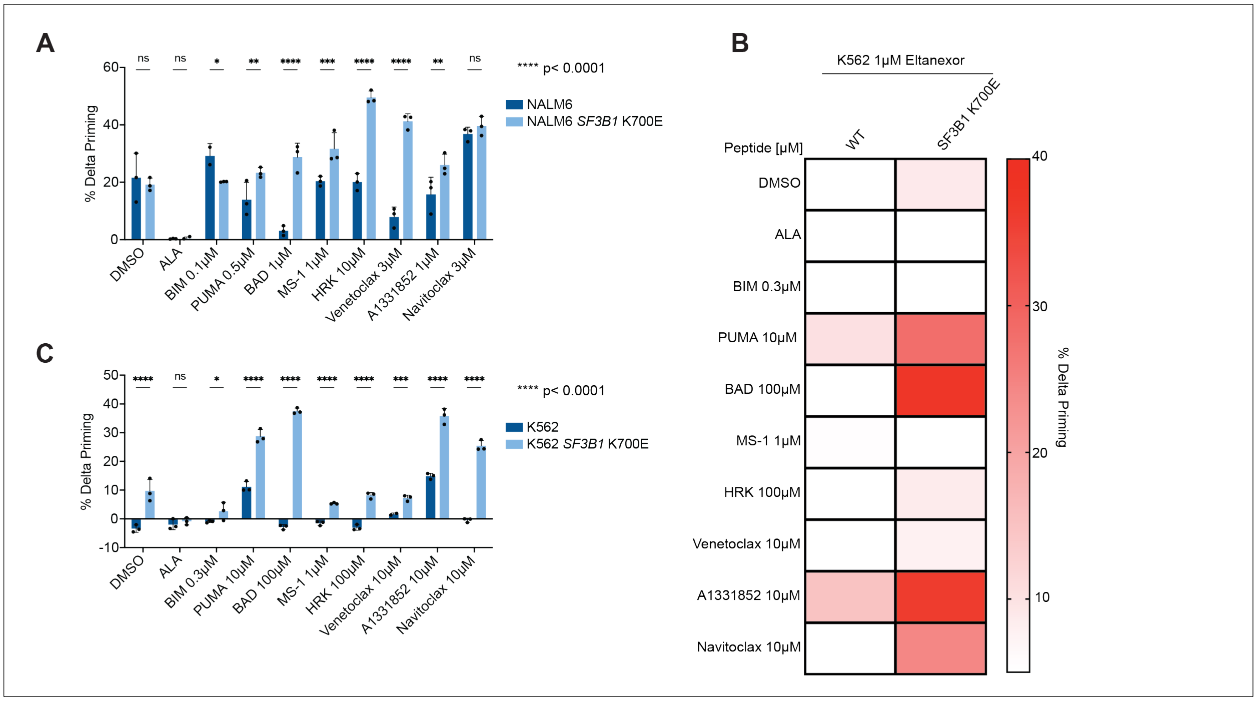

Supplementary Figure S2. ***SF3B1* mutations have BCLXL dependence.** (**A**) Delta priming responses of the indicated BH3 peptides and mimetics in NALM6 SF3B1 WT and mutant cells (**B)** K562 SF3B1 WT and mutant cells after 16 hours of 1µM eltanexor treatment compared to DMSO. **** p<0.0001 (n= 3 replicates). (**C**) BH3 profiling of K562 SF3B1 WT and mutant cells after 16 hours of eltanexor treatment in a heatmap with the delta priming responses.

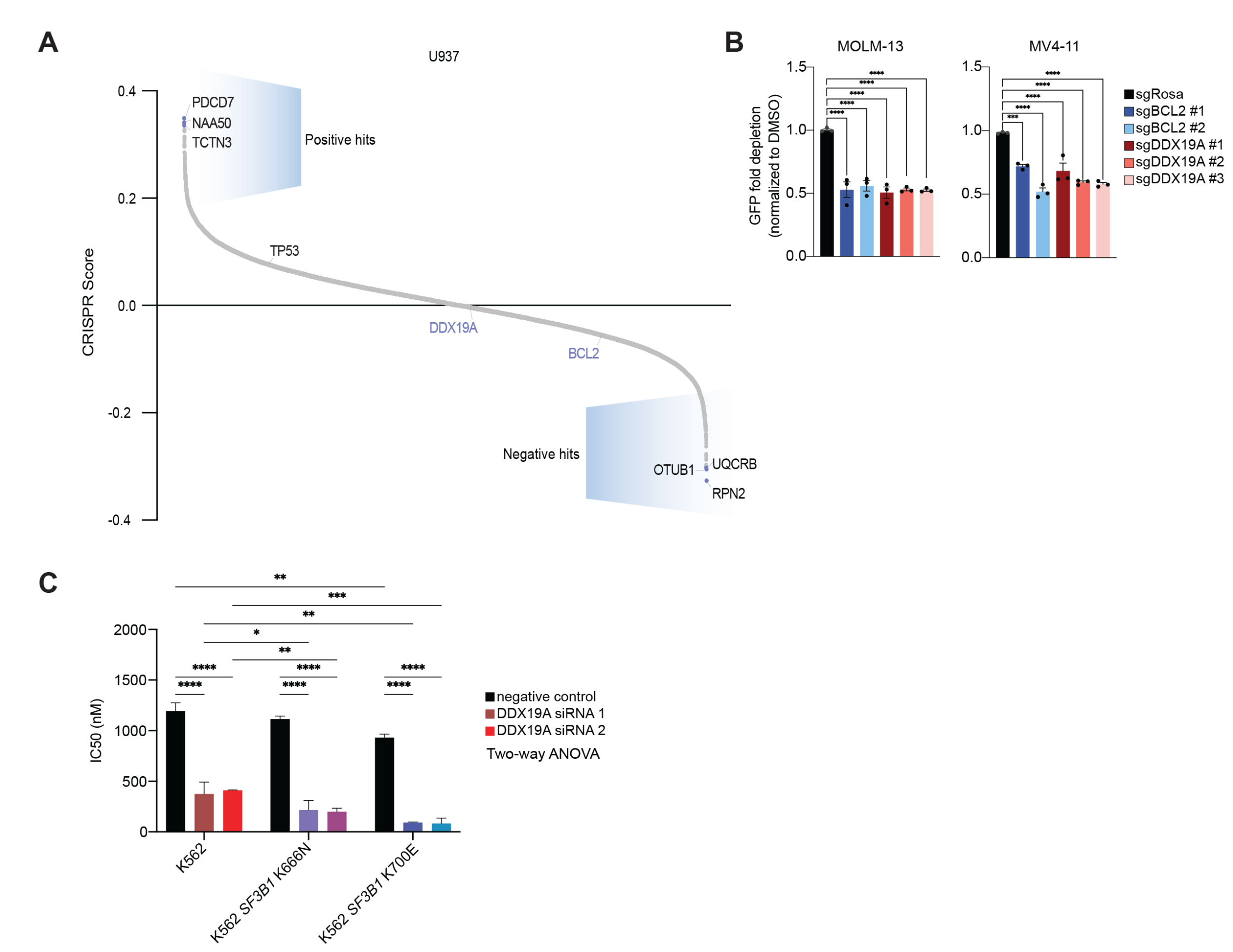

Supplementary Figure S3. CRISPR Screen identifies BCL2 and DDX19A associated with XPO1 inhibition. (**A**) Genome-wide CRISPR screen of U937 cells. Colored labels indicate genes involved in BCL pathway. (**B**) Validation of BCL2 and DDX19A sgRNAs into MOLM-13 and MV4-11 cells (n=3 replicates, mean + SEM). (**C**) IC50 values of DDX19A knockdown cells treated with eltanexor. Two-way ANOVA (n=3 replicates).

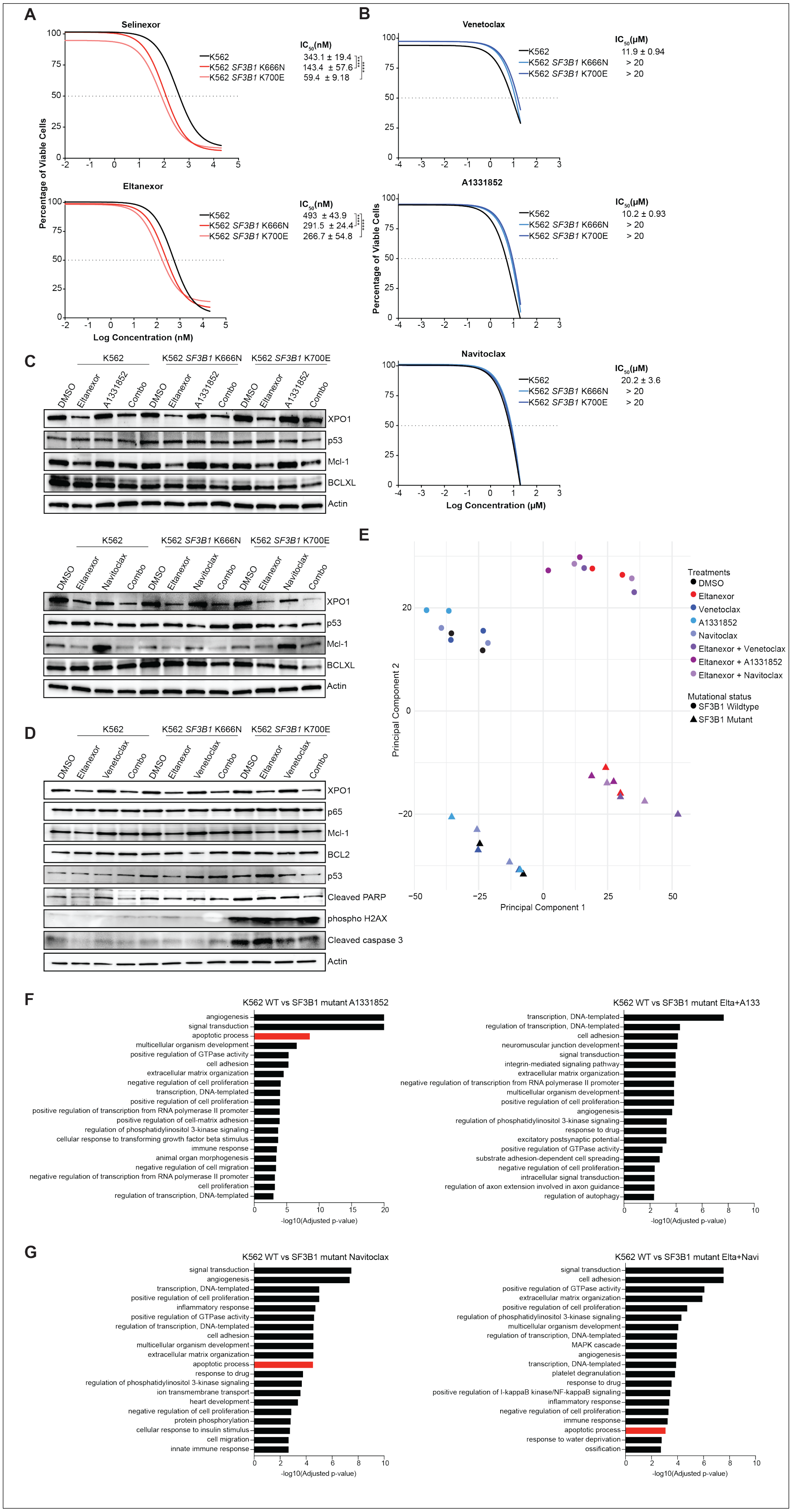
A
Selinexor
Eltanexor
Percentage of Viable Cells
Log Concentration (nM)
K562
K562 SF3B1 K666N
K562 SF3B1 K700E
IC50(nM)
343.1 ± 19.4
143.4 ± 57.6
59.4 ± 9.18
493 ± 43.9
291.5 ± 24.4
266.7 ± 54.8
B
Venetoclax
A1331852
Navitoclax
IC50(μM)
11.9 ± 0.94
> 20
> 20
10.2 ± 0.93
> 20
> 20
20.2 ± 3.6
> 20
> 20
Percentage of Viable Cells
Log Concentration (μM)
C
K562
K562 SF3B1 K666N
K562 SF3B1 K700E
DMSO
Eltanexor
A1331852
Combo
Navitoclax
XPO1
p53
Mcl-1
BCLXL
Actin
D
DMSO
Eltanexor
Venetoclax
Combo
XPO1
p65
Mcl-1
BCL2
p53
Cleaved PARP
phospho H2AX
Cleaved caspase 3
Actin
E
Principal Component 1
Principal Component 2
Treatments
DMSO
Eltanexor
Venetoclax
A1331852
Navitoclax
Eltanexor + Venetoclax
Eltanexor + A1331852
Eltanexor + Navitoclax
Mutational status
SF3B1 Wildtype
SF3B1 Mutant
F
K562 WT vs SF3B1 mutant A1331852
angiogenesis
signal transduction
apoptotic process
multicellular organism development
positive regulation of GTPase activity
cell adhesion
extracellular matrix organization
negative regulation of cell proliferation
transcription, DNA-templated
positive regulation of cell proliferation
positive regulation of transcription from RNA polymerase II promoter
positive regulation of cell-matrix adhesion
regulation of phosphatidylinositol 3-kinase signaling
cellular response to transforming growth factor beta stimulus
immune response
animal organ morphogenesis
negative regulation of cell migration
negative regulation of transcription from RNA polymerase II promoter
cell proliferation
regulation of transcription, DNA-templated
-log10(Adjusted p-value)
K562 WT vs SF3B1 mutant Elta+A133
transcription, DNA-templated
regulation of transcription, DNA-templated
cell adhesion
neuromuscular junction development
signal transduction
integrin-mediated signaling pathway
extracellular matrix organization
negative regulation of transcription from RNA polymerase II promoter
multicellular organism development
positive regulation of cell proliferation
angiogenesis
regulation of phosphatidylinositol 3-kinase signaling
response to drug
excitatory postsynaptic potential
positive regulation of GTPase activity
substrate adhesion-dependent cell spreading
negative regulation of cell proliferation
intracellular signal transduction
regulation of axon extension involved in axon guidance
regulation of autophagy
-log10(Adjusted p-value)
G
K562 WT vs SF3B1 mutant Navitoclax
signal transduction
angiogenesis
transcription, DNA-templated
positive regulation of cell proliferation
inflammatory response
positive regulation of GTPase activity
regulation of transcription, DNA-templated
cell adhesion
multicellular organism development
extracellular matrix organization
apoptotic process
response to drug
regulation of phosphatidylinositol 3-kinase signaling
ion transmembrane transport
heart development
negative regulation of cell proliferation
protein phosphorylation
cellular response to insulin stimulus
cell migration
innate immune response
-log10(Adjusted p-value)
K562 WT vs SF3B1 mutant Elta+Navi
signal transduction
cell adhesion
positive regulation of GTPase activity
extracellular matrix organization
positive regulation of cell proliferation
regulation of phosphatidylinositol 3-kinase signaling
multicellular organism development
regulation of transcription, DNA-templated
MAPK cascade
angiogenesis
transcription, DNA-templated
platelet degranulation
response to drug
positive regulation of I-kappaB kinase/NF-kappaB signaling
inflammatory response
negative regulation of cell proliferation
immune response
apoptotic process
response to water deprivation
ossification
-log10(Adjusted p-value)

Supplementary Figure S4. SF3B1 mutant cells show increased synergy with the combination of XPO1 inhibitor and BCL2. (**A**) Dose-response curves of K562 cells treated with XPO1 inhibitors, eltanexor and selinexor, for 72 hours (n=4 replicates). Two-way ANOVA. (**B**) Dose-response curves of K562 cells treated with BCL inhibitors, venetoclax, A-1331852, and navitoclax, for 72 hours (n=4 replicates). (**C**) Western blot analysis of XPO1 and BCL targets after treatment with vehicle, 200nM eltanexor, 1μM A-1331852, and combination of 200nM eltanexor and 1μM A-1331852 (top) and vehicle, 200nM eltanexor, 1μM navitoclax, and the combination of 200nM eltanexor and 1μM navitoclax (bottom) for 24 hours. (**D**) Western blot of XPO1 downstream targets and apoptosis targets in K562 cells after 24 hours of exposure with vehicle, 200nM eltanexor, 1μM venetoclax, and the combination. (**E**) PCA plot of K562 WT and SF3B1 mutant cells with eight treatment groups: vehicle (DMSO), eltanexor, venetoclax, A1331852, navitoclax, eltanexor and venetoclax, eltanexor and A1331852, and eltanexor and navitoclax (n=2 replicates). (**F**) GO pathway enrichment analysis of the differentially expressed genes between wildtype and SF3B1 mutant with the A1331852 treatment (left) and eltanexor with A1331852 treatment (right). (**G**) GO pathway enrichment analysis of the differentially expressed genes between wildtype and SF3B1 mutant cells with the navitoclax treatment (left) and eltanexor with navitoclax treatment (right).

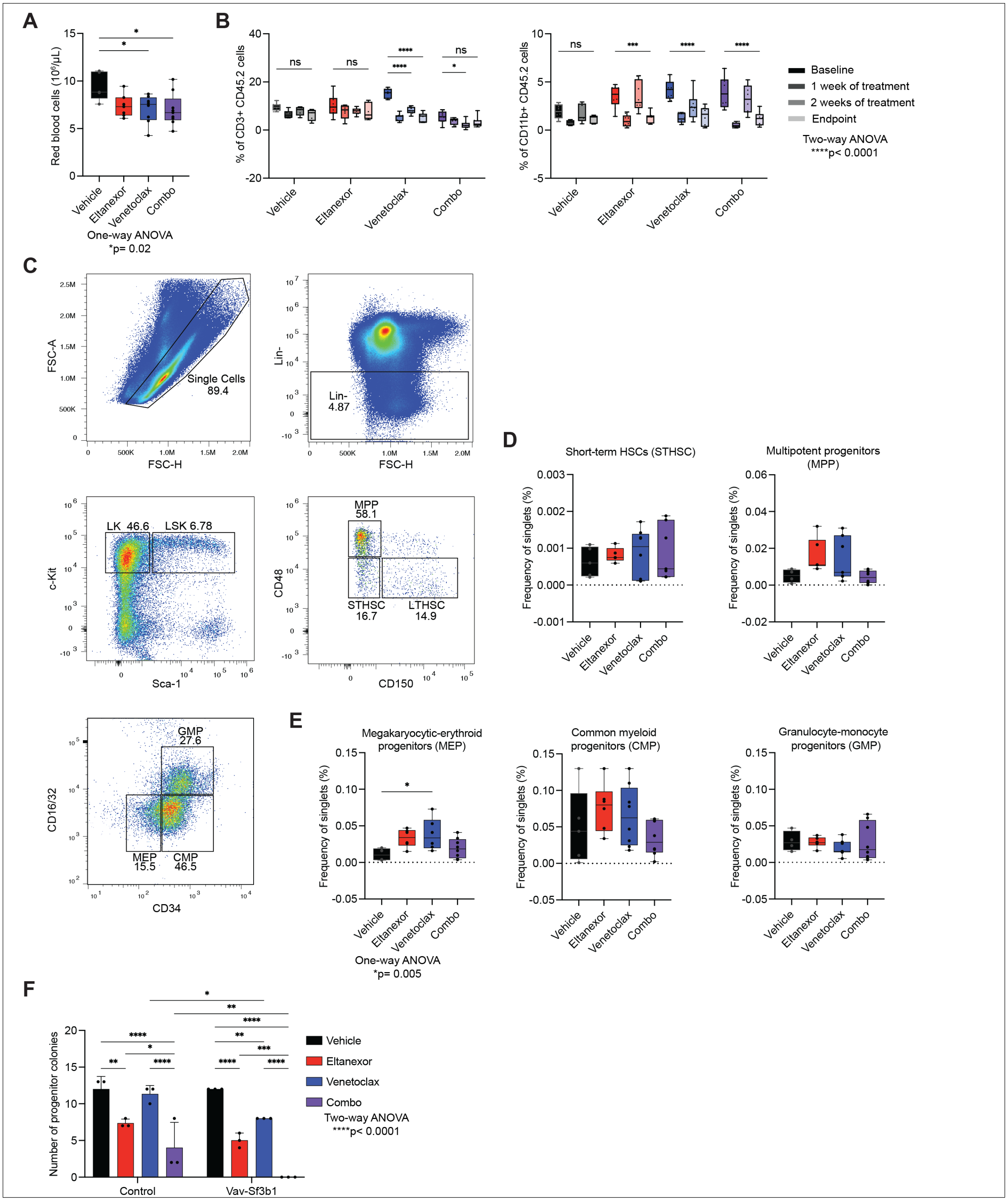

Supplementary Figure S5. Combination of XPO1 inhibitor and BCL2 inhibitor, venetoclax, show trend towards decrease in hematopoietic and stem cell compartment of the Sf3b1 mutant cells. (**A**) Effect of eltanexor, venetoclax, and the combination on red blood cells at the

endpoint of experiments. Data is shown as mean ± standard deviation, One-way ANOVA p=0.02. (**B**) Violin plot of CD3+ (T-cells) and Cdllb+ (macrophages and dendritic cells) of the CD45.2 compartment in peripheral blood before treatment, one week of treatment, two weeks of treatment, and at endpoint. Data is shown as mean ± standard deviation, two-way ANOVA ****p<0.0001. (**C**) Gating strategy for analysis of hematopoietic stem and progenitors cells using FlowJo v10. (**D**) Percentage of short-term hematopoietic stem cells (STHSC) and multipotent progenitors (MPP) in SF3B1 mutant cells. (**E**) Percentage of megakaryocytic-erythrocyte progenitors (MEP), common myeloid progenitors (CMP), and granulocyte-macrophage progenitors (GMP) in the CD45.2+ compartment, One-way ANOVA p=0.005. Combo= eltanexor and venetoclax. (**F**) Colony formation of HSPCs treated with vehicle, eltanexor, venetoclax, or the combination. Data is shown as mean ± standard deviation.

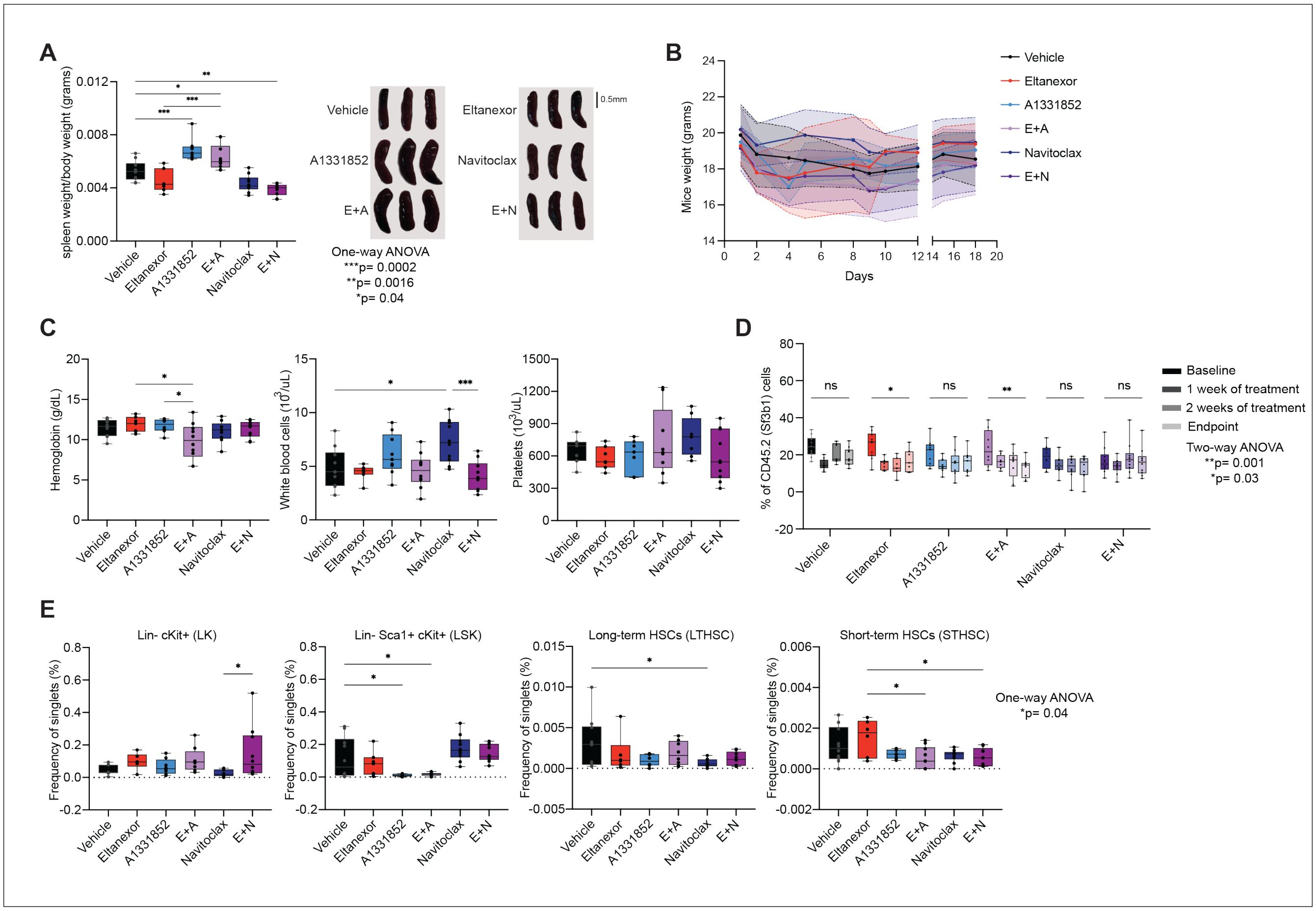

Supplementary Figure S6. Combination of XPO1 inhibitor and BCLXL inhibitors show trend towards decrease in Sf3b1 mutant cells. (**A**) Bar chart of reduced size and weight of spleen in combination of eltanexor and A1331852 and increased spleen size in combination of eltanexor and navitoclax treated mice with representative images of the spleens. Scale bar; 0.5mm. One-way ANOVA ***p=0.0002, **p=0.0016, *p=0.04.(**B**) Comparison of the body weight between the treatment groups of vehicle, eltanexor (10 mg/kg), A1331852 (25 mg/kg), eltanexor + A1331852, navitoclax (25mg/kg) and eltanexor + navitoclax. Data is shown as mean ± standard deviation. (**C**) Effect of eltanexor, A1331852, navitoclax and each of the combinations on hematological parameters (hemoglobin, white blood cells, and platelets) at the endpoint of experiments. Data is shown as mean ± standard deviation, one-way ANOVA. (**D**) Violin plot of CD45.2+ compartment in peripheral blood before treatment, one week of treatment, two weeks of treatment, and at endpoint. Data is shown as mean ± standard deviation, two-way ANOVA. (**E**) Percentage of $Lin^{-}cKit^{+}$ (LK), Lin^{-} $Sca1^{+}$ $cKit^{+}$ (LSK), and long-term hematopoietic stem cells (LTHSC) and short-term hematopoietic stem cells (STHSC) in CD45.2+ compartment, one-way ANOVA. E+A= eltanexor and A1331852, E+N= eltanexor and navitoclax.
